# Qualitative and Quantitative Imaging Evaluation of Renal Cell Carcinoma Subtypes with Grating-based X-ray Phase-contrast CT

**DOI:** 10.1038/srep45400

**Published:** 2017-03-31

**Authors:** Margarita Braunagel, Lorenz Birnbacher, Marian Willner, Mathias Marschner, Fabio De Marco, Manuel Viermetz, Susan Notohamiprodjo, Katharina Hellbach, Sigrid Auweter, Vera Link, Christine Woischke, Maximilian F. Reiser, Franz Pfeiffer, Mike Notohamiprodjo, Julia Herzen

**Affiliations:** 1 Institute of Clinical Radiology, Ludwig-Maximilian-University Hospitals Munich, Munich, Germany; 2 Lehrstuhl für Biomedizinische Physik, Physik-Department & Institut für Medizintechnik, Technische Universität München, Garching, Germany; 3 Department of Pathology, Ludwig-Maximilian-University Hospitals Munich, Munich, Germany; 4 Department of Radiology, University Hospital Tübingen, Germany

## Abstract

Current clinical imaging methods face limitations in the detection and correct characterization of different subtypes of renal cell carcinoma (RCC), while these are important for therapy and prognosis. The present study evaluates the potential of grating-based X-ray phase-contrast computed tomography (gbPC-CT) for visualization and characterization of human RCC subtypes. The imaging results for 23 *ex vivo* formalin-fixed human kidney specimens obtained with phase-contrast CT were compared to the results of the absorption-based CT (gbCT), clinical CT and a 3T MRI and validated using histology. Regions of interest were placed on each specimen for quantitative evaluation. Qualitative and quantitative gbPC-CT imaging could significantly discriminate between normal kidney cortex (54 ± 4 HUp) and clear cell (42 ± 10), papillary (43 ± 6) and chromophobe RCCs (39 ± 7), p < 0.05 respectively. The sensitivity for detection of tumor areas was 100%, 50% and 40% for gbPC-CT, gbCT and clinical CT, respectively. RCC architecture like fibrous strands, pseudocapsules, necrosis or hyalinization was depicted clearly in gbPC-CT and was not equally well visualized in gbCT, clinical CT and MRI. The results show that gbPC-CT enables improved discrimination of normal kidney parenchyma and tumorous tissues as well as different soft-tissue components of RCCs without the use of contrast media.

Most renal lesions are incidentally detected in ultrasound or computed tomography (CT) often presenting without clinical symptoms. Thereby, renal cell carcinoma (RCC) account for 2–3% of all adult cancers worldwide^1^. The common subtypes of sporadic RCC are clear cell (ccRCC; 70–85%), papillary (pRCC; 7–15%) and chromophobe RCC (chrRCC; 5–10%)^2^. Each RCC subtype has a different prognosis, metastatic rate and differs in the response to targeted therapies^3^. Differentiation of renal lesions is limited for non-enhanced CT due to its low soft-tissue contrast^4^. The use of contrast agents improves the detection and discrimination of different RCC subtypes using multiphasic CT^4^,^5^,^6^ and magnetic resonance imaging (MRI)^7^,^8^. Iodine-based contrast agents can cause acute renal failure, anaphylactic reactions or thyrotoxic crisis while gadolinium-based contrast media can cause nephrogenic systemic fibrosis. Contraindications for MRI are cardiac pacemakers and patients with claustrophobia.

For preoperative histological diagnosis percutaneous renal tumor biopsies are often used, but potential postbioptic complications^9^ lead to limited acceptance.

Phase-contrast computed tomography (PC-CT) is a promising new X-ray-based imaging method. In contrast to conventional CT, where the attenuation of X-rays is measured, the refraction of the X-ray beam that occurs when the beam passes through tissue is visualized in phase-contrast imaging^10^. Different techniques can be used to derive the phase-contrast signal, e.g. propagation-based, analyzer-based or crystal-interferometer-based methods, however, these approaches face some limitations with respect to the implementation into a clinical environment^11^. Another method is the grating interferometry, which simultaneously yields three complimentary signals – the conventional attenuation, a phase-contrast and a dark-field image. Pfeiffer *et al*.^12^ showed that conventional polychromatic X-ray sources can be used for phase-contrast imaging with a three gratings interferometer, which is a prerequisite for clinical application. With this approach, previous studies showed an increased soft-tissue contrast, e.g. in breast specimens^13^,^14^,^15^, atherosclerotic plaques^16^,^17^, liver lesions^18^, murine kidneys with and without renal ischemia^19^ and other soft-tissue components^20^,^21^.

The purpose of this *ex vivo* study was to evaluate the potential of grating-based PC-CT (gbPC-CT) imaging for the visualization of tumor architecture and for the characterization of different RCC subtypes in comparison to attenuation-based CT and MRI and to correlate the results with histopathology as the standard of reference.

## Results

### Normal kidney parenchyma

**Figure 1:**
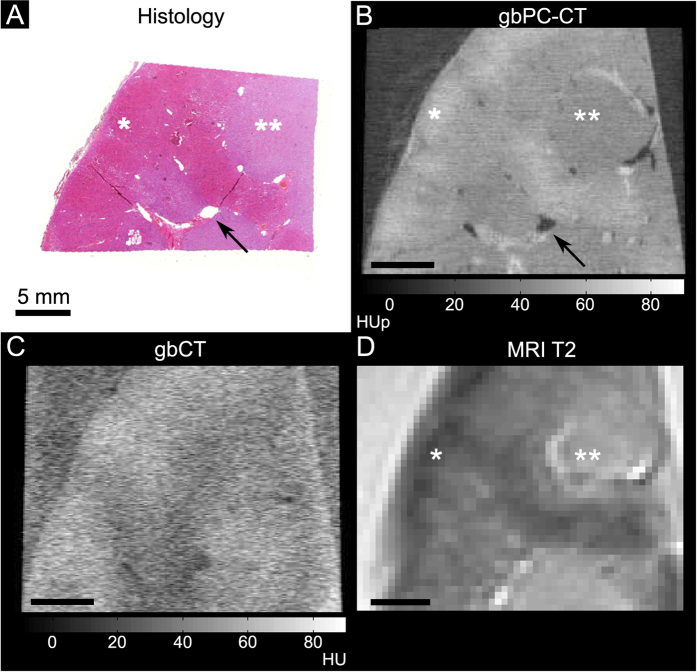
Normal human kidney sample imaged with phase-contrast CT, grating-based attenuation-based CT, T2-w magnetic resonance imaging and histologic slice (coronal slice). Good visual agreement between the histology ((**A**); HE-staining) and grating-based phase-contrast CT ((**B**); gbPC-CT) showed a higher soft-tissue contrast and a clear discrimination of renal vessels (arrow) and between the cortex (*) with higher and the medulla (**) with lower phase-contrast signal with good comparison to T2-w magnetic resonance imaging (MRI) (**D**). Imaging with grating-based attenuation-based CT (gbCT) from the same setup (**C**) had an obvious lower soft-tissue contrast.

GbPC-CT of normal human kidney allowed a qualitative and quantitative differentiation between cortex (54 ± 4 HUp) and medulla (44 ± 3 HUp; *p* < 0.05) and showed a good visual agreement to T2w-MRI and histologic slices (Fig. 1). Vessels were clearly distinguished from kidney parenchyma. In gbCT (Fig. 1) and clinical CT (clinCT; not shown), discrimination of cortex or medulla was not possible.

### Qualitative Analysis of Renal Cell Carcinomas

**Figure 2:**
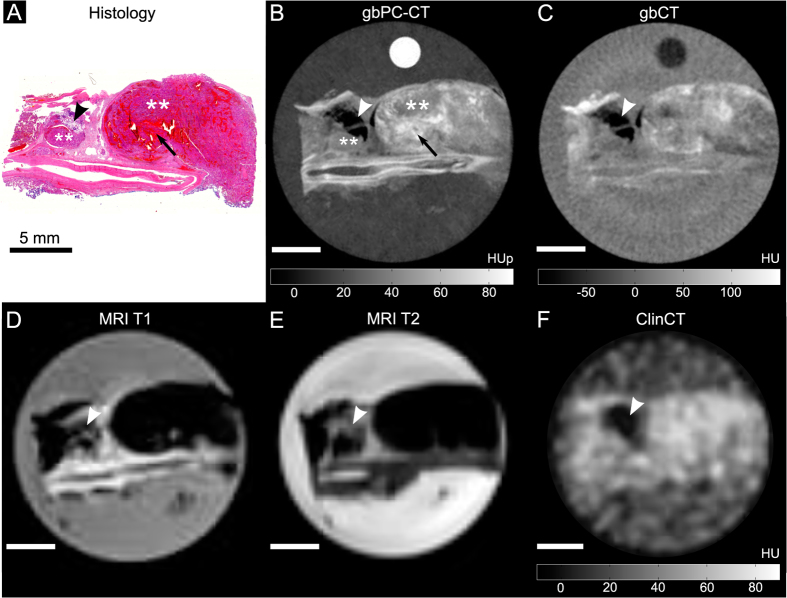
Clear cell renal cell carcinoma sample imaged with phase-contrast CT, grating-based and clinical CT and MRI with corresponding histological slice. Good visual agreement between histological slice ((**A**); HE-staining) and corresponding phase-contrast CT image ((**B**); gbPC-CT) with distinct differentiation of large and small tumor nodules with low signal (**), diffuse intratumoral bleedings (arrow), as well as a fatty area (arrowhead) and a large vessel at the bottom of the slice. GbPC-CT (**B**) showed a superior visualization of tumor boundaries and different tumor nodules (**) than with gbCT (**C**) and clinical CT (**F**), which could only detect fat (arrowhead) and soft-tissue (area of hyperdensity). GbPC-CT imaging (**A**) showed a better depiction of the tumor components like intratumoral bleeding than in MRI images (**D** and **E**) due to susceptibility artifacts.

**Figure 3:**
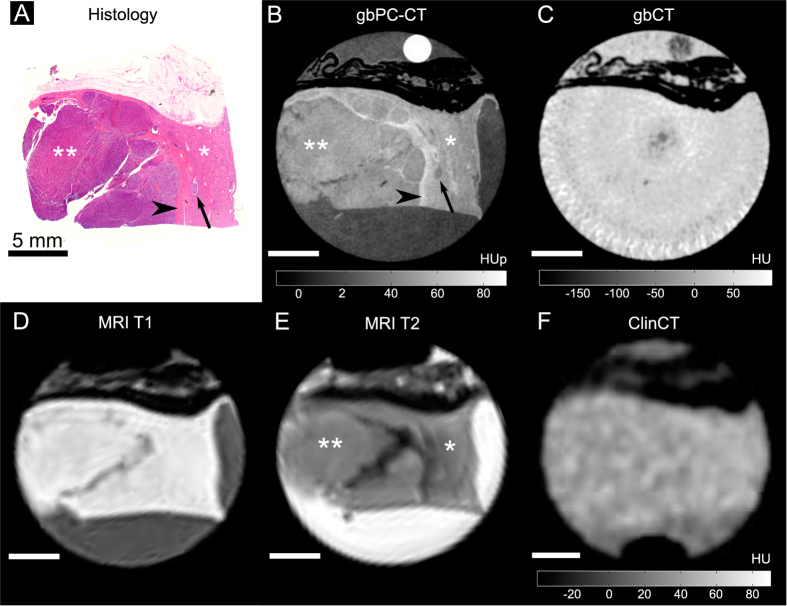
Papillary renal cell carcinoma sample imaged with phase-contrast CT, grating-based and clinical CT and MRI with corresponding histological slice. A perfect match of the histological slice (**A**) and the phase-contrast (gbPC-CT) image (**B**) was seen with a clear discrimination of the normal cortex (*) with a higher phase-contrast signal and the homogeneous tumor area (**) with lower signal in gbPC-CT. Additionally, gbPC-CT could depict the pseudocapsule surrounding the tumor with a higher signal (arrowhead) than cortex, a micrometastasis in the cortex (arrow) as well as small linear fibrous strands. In grating-based CT (gbCT) (**C**) and clinical CT (**F**), only perirenal fat was visible (hypodense), soft-tissue components could not be differentiated. When correlating gbPC-CT (**B**) with MR images (**D**,**E**), a superior discrimination of tumor and normal kidney is seen in the phase-contrast image.

**Figure 4:**
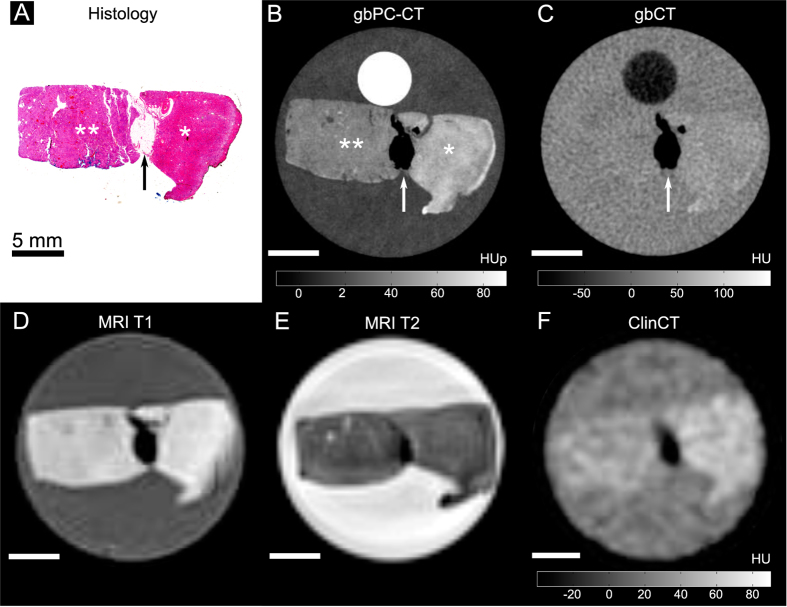
Chromophobe renal cell carcinoma sample imaged with phase-contrast CT, grating-based and clinical CT and MRI with corresponding histological slice. A good correlation was seen between histology (**A**) and phase-contrast imaging (gbPC-CT; (**B**)) for clear detection of normal kidney (*) with significant higher signal (*p* < 0.05) and the homogeneous tumor with lower signal (**). In the middle, a stripe of fat with low signal (arrow) is clearly visualized with all imaging techniques. In comparison to gbPC-CT, grating-based CT (gbCT) (**C**), clinical CT (**F**) and MRI (**D**,**E**) could only show a marginal visible difference between normal and tumorous tissue.

Qualitative image analysis showed that gbPC-CT imaging allowed a reliable differentiation of tumorous tissue of ccRCCs, pRCCs and chrRCCs from normal renal cortex (Figs 2–4). Tumorous tissue showed visually lower phase-contrast intensity, a loss of normal cortical or medullar structure and absent normal vessels in comparison to normal kidney.

The sensitivity was 100% for detection of tumor areas on gbPC-CT and MRI as verified by histology. Tumor boundaries could be differentiated from normal kidney, and showed excellent visual agreement with histological slices. Images of attenuation-based CTs showed a significantly lower sensitivity for detection and discrimination of tumor suspicious areas from normal parenchyma (gbCT: 50%; clinCT: 40%). A clear delineation of tumor boundaries was not possible (Figs 2–4).

**Figure 5:**
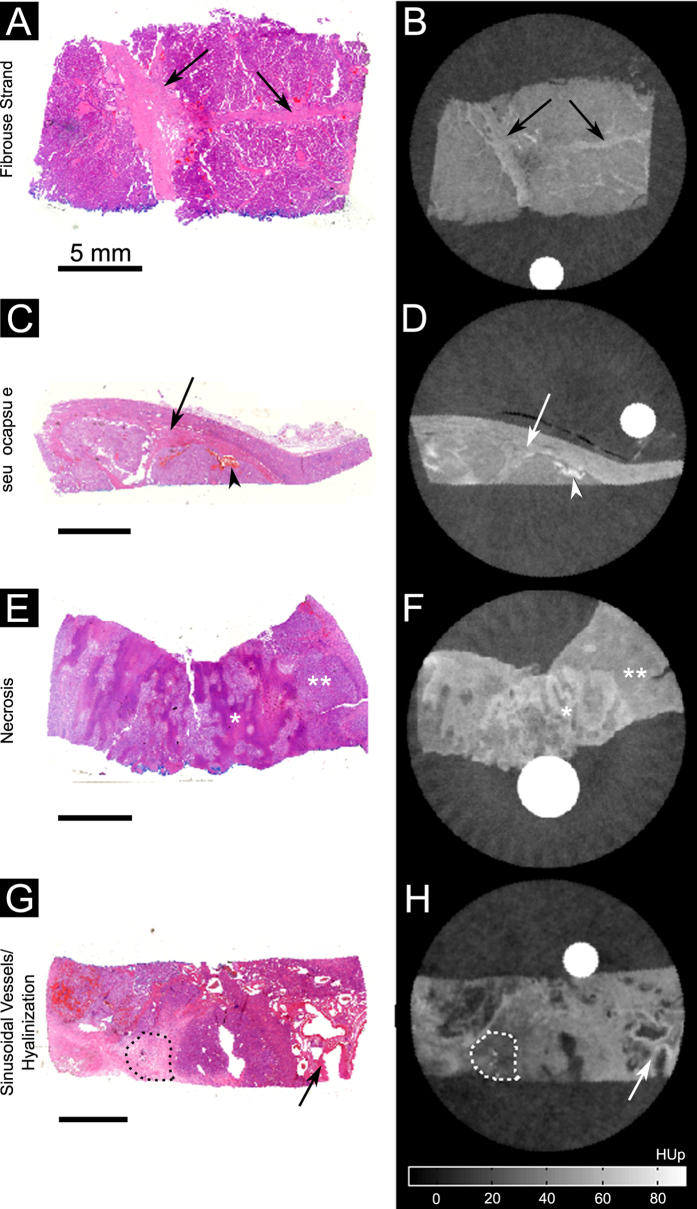
Visual comparison between histological slices and grating-based PC-CT images. Histological slices (HE-staining) (**A**,**C**,**E**,**G**) showed a good visual agreement with phase-contrast images (**B**,**D**,**F**,**H**) for visualization of different tumor tissue components (arrows) like fibrous strands (**A**,**B**), pseudocapsule (arrow) and hemorrhage (arrowhead) (**C**,**D**), diffuse necrotic areas with higher signal (*) and tumor tissue with lower phase-contrast signal (**) (**E**,**F**) as well as sinusoidal-vessels (arrow) and hyalinization (dotted line) (**G**,**H**).

GbPC-CT showed a high sensitivity for detection of soft-tissue components (Table 1, Fig. 5). Small dot-like microbleeding and diffuse hemorrhage showed a high signal and a wide distribution throughout the tumorous tissue. Fibrous strands and pseudocapsules were detected as linear contrast-rich bands that can be found throughout the low-signal tumorous tissue and surrounding the tumor boundaries, respectively (Fig. 5). These tissue components could not be visualized with gbCT and clinCT (Table 1). Areas of necrosis showed a higher and hyalinization a lower signal in comparison to tumorous tissue (Fig. 5), with a lower detection rate in gbPC-CT, gbCT and MRI in comparison to histology and with the lowest sensitivity in clinCT (Table 1).

Small dot-like calcifications (microcalcification) were clearly visualized in gbPC-CT. Larger calcifications showed the highest soft-tissue signals with small surrounding artifacts and an excellent visualization in gbPC-CT and gbCT and a lower detection rate in clinCT (Table 1). Fat depositions were clearly visualized in all imaging methods due to the lowest signals (Figs 2–4).

**Figure 6:**
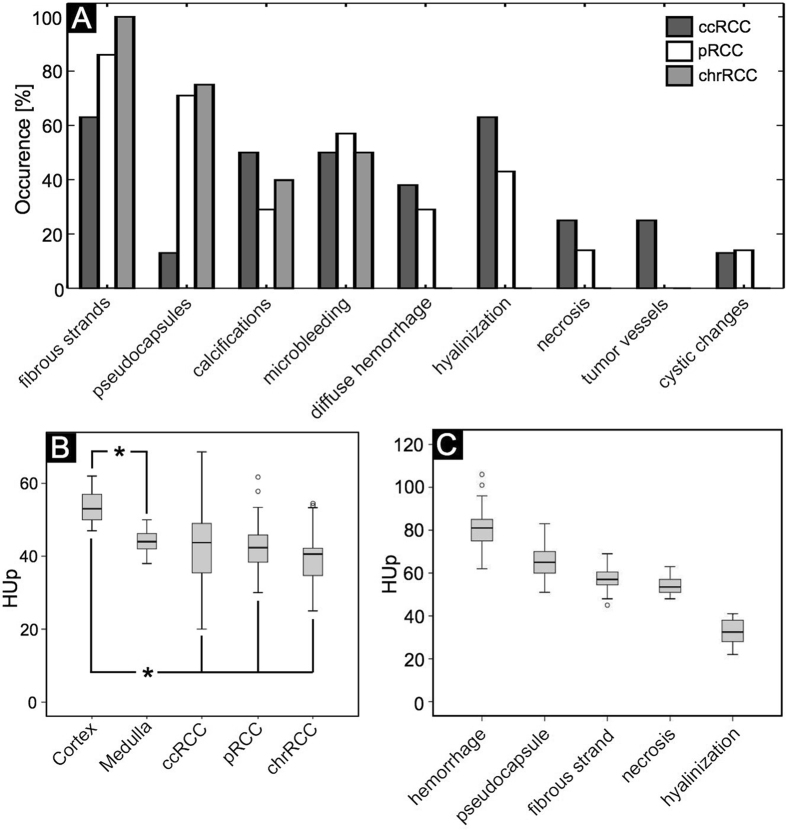
Occurrence of tissue components and quantification of different tumors and soft-tissue components in renal cell carcinoma subtypes. Data obtained from gbPC-CT in good correlation with histology. (**A**) Bar graphs visualizing the percentaged occurrence visualized in grating-based phase-contrast CT in agreement with histologic slices of various tumor tissue components like fibrous strands, pseudocapsules, calcifications, hyalinization, necrotic areas and sinusoidal-vessels in the different renal cell carcinoma subtypes. (ccRCC = clear cell; pRCC = papillary; chrRCC = chromophobic renal cell carcinoma). (**B**) In grating-based phase-contrast CT, the cortex of normal human kidney showed significant higher HUp-values than medulla (*means *p* < 0.05). A significant difference of quantitative phase-contrast values was seen between cortex and the tumor tissue of all renal cell carcinoma (RCC) subtypes (*means *p* < 0.05, respectively). (**C**) Significant quantitative difference of HUp-values in phase-contrast imaging was seen between the different tumor components. The highest values were seen for hemorrhage, lower for pseudocapsule, fibrosis and necrosis and the lowest in hyalinization areas with a significant difference from each other (*p* < 0.05, respectively).

In good agreement with the histological examination, most ccRCCs (75%) had a heterogeneous appearance in gbPC-CT with detection of hyalinization, necrotic areas, local and diffuse hemorrhage, small calcifications, sinusoid-like tumor vessels and cystic changes. Papillary RCC and chrRCCs were more homogeneous (71% and 100%, respectively) with linear, contrast-rich bands in the low-signal tumor tissue corresponding to fibrous strands and pseudocapsules and displayed fewer calcifications than ccRCC. Diffuse hemorrhage, hyalinization, necrosis or cystic changes were not seen in chrRCCs. The occurrence of the different tissue components in each RCC subtype is visualized in Fig. 6.

No intratumoral tissue components like fibrous strands, pseudocapsules or microbleedings were visualized with gbCT and clinCT. About half of the ccRCC samples showed a hypodense and inhomogeneous tissue appearance with large calcifications (3/8) and diffuse hemorrhage (2/8) detected in clinCT and gbCT, the other samples showed hyperdense tissues without a possible differentiation from normal kidney. Homogeneous hypodense tissues were detected in pRCC and chrRCC subtypes in gbCT (4/7 and 2/5, respectively) and clinCT (3/7 and 2/5, respectively) with large calcification in one chrRCC sample.

### Quantitative Analysis of Renal Cell Carcinomas

Tumorous tissue of ccRCCs (n = 8), pRCCs (n = 7) and chrRCCs (n = 5) could be differentiated from normal renal cortex (n = 6) due to a significantly lower signal (Fig. 6 B; p < 0.05). A quantitative differentiation of renal medulla and tumorous tissue and a differentiation between ccRCC and pRCC or chrRCC was not possible (Fig. 6B).

Variable tumor tissue components were detected in gbPC-CT due to significant different signal intensities. Hemorrhage showed significantly higher phase-contrast signal (80 ± 9 HUp) than pseudocapsule (66 ± 8 HUp), fibrotic strands (57 ± 5 HUp), and necrosis (54 ± 5 HUp). Compared to these, hyalinization showed significantly lower signal intensity (p < 0.05, respectively; Fig. 6). Calcifications with the highest densities (280 ± 199 HUp) as well as fat (−60 ± 10 HUp) with the lowest density could be clearly differentiated from soft tissue. Compared to MRI, where calcifications and microbleedings cannot be separated due to same susceptibility artifacts, gbPC-CT showed a good discrimination between hemorrhage (80 ± 9 HUp) and larger calcifications (>290 HUp) with small artifacts.

## Discussion

Our results of this *ex vivo* feasibility study indicate that grating-based X-ray phase-contrast imaging is able to qualitatively and quantitatively discriminate normal kidney from renal cell carcinomas superior to absorption-based CTs without application of contrast media. Our findings are in good agreement with a recent study^19^ showing an improved evaluation of normal and ischemic renal parenchyma in murine kidneys using synchrotron-based phase-contrast imaging. Compared to previous studies^22^,^23^, our measurements for cortex and medulla of healthy human renal samples were elevated, which may occur due to longer storage times (see Willner *et al*.^23^).

In terms of depiction and quantitative differentiation of fine intratumoral structures we could demonstrate that gbPC-CT showed a good agreement to histopathology and is superior to MRI and also to unenhanced absorption-based CTs independent from the effective pixel size (clinCT: 400 vs. gbCT: 100 μm).

The detection and characterization of incidentally imaged renal lesions on unenhanced CT remains difficult due to the method’s low soft-tissue contrast^4^. Additionally, unenhanced CT values of different RCC subtypes showed divergent measurements^4^,^24^. Contrast enhanced multiphasic CT and MRI images are used in clinical routine to diagnose RCC and to define the subtype^5^,^8^. The accuracy of discriminating ccRCC from papillary and chromophobe RCC was reported with 85% and 84% in multiphasic CT^4^. Also differentiation of malignant subtypes is getting more difficult, when tumor size decreases^25^. But both approaches, CT and MRI, face significant limitations. In this context, gbPC-CT may improve diagnostics without any use of contrast agents in future.

In good agreement to histology, clear cell carcinomas showed a trend towards a more heterogeneous appearance with diffuse hemorrhages, hyalinization, necrosis and sinusoidal vessels in contrast to pRCC and chrRCC in gbPC-CT. Papillary RCC showed a higher detection of fibrous strands, pseudocapsules and microbleedings as well as diffuse hemorrhage and cystic changes. Chromophobe RCCs were the most homogeneous tumors with fibrous strands and microbleeding without detection of hyalinization or necrosis. This detailed information obtained with gbPC-CT may further be used in addition to CT or MRI images to elevate the accuracy of diagnosing RCC subtypes in the future. However, a separation of RCC subtypes based only on quantitative HUp-values cannot be achieved.

Histology has a significantly higher spatial resolution than gbPC-CT, clinCT or MRI, and numerous additional tests like immunochemistry are available. For example, a histological diagnosis of chromophobe tumor can only be made after Hale’s staining.

Limitation of this study is that gbPC-CT is currently available only in preclinical settings. Furthermore, the *ex vivo* RCC samples in this study were scanned without contrast media in the PC-CT setup, clinical CT and MRI, which could have decreased the sensitivity. Finally, Hounsfield units of attenuation-based CTs are energy-dependent^20^ and, therefore, a direct quantitative comparison between the two CTs (gbCT acquired at 40 and clinCT at 120 kVp) cannot be performed. Currently, the field-of-view of gbPC-CT is limited by the size of the available gratings while a previous study by Willner *et al*.^26^ could demonstrate that the grating-based method can also work at higher, clinically relevant energies.

**Figure 7:**
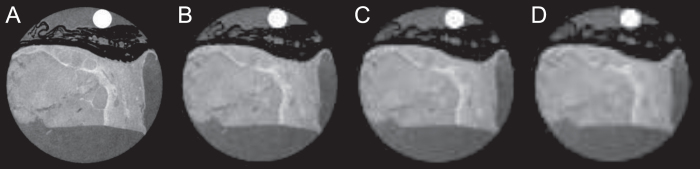
Influence of the image resolution on the gbPC-CT imaging results. GbPC-CT slice of a papillary renal cell carcinoma sample from Fig. 3 shown at different effective resolutions: (**A**) 100 × 100 μm^2^, (**B**) 300 × 300 μm^2^, (**C**) 400 × 400 μm^2^, (**D**) 500 × 500 μm^2^. Different effective resolutions were obtained through retrospective binning of the original data.

The resolution of the gbPC-CT in this study was significantly higher than the one typically used in clinical imaging. Such a high resolution has no limitation for histopathological workup scans, but it is not compatible with clinical patient imaging. In general, it can be expected that also for clinically relevant resolutions, phase-contrast imaging will still provide a superior contrast between different soft tissue subtypes compared to the conventional attenuation imaging^27^,^28^. However, at lower image resolution the border delineation between e.g. healthy and tumorous tissue will be more challenging. This effect is highlighted in Fig. 7, where the sample from Fig. 3 is presented at different effective resolutions, obtained through retrospective image binning. While it is possible to tell the difference between healthy and tumorous tissue even at 500 × 500 μm^2^ resolution, the exact border outline is not as obvious as it is in the case of 100 × 100 μm^2^ resolution.

The high resolution used in this study also implied that the dose delivered to the samples is far beyond any clinical CT scan. The dose level in this study is better comparable to a microCT. Our result correlates well with earlier microCT studies that showed even at an elevated dose level, absorption contrast was not able to yield the same soft tissue contrast as obtained through phase-contrast imaging^16^,^19^,^29^.

All in all, this proof-of-principle study demonstrates that gbPC-CT has the potential to provide diagnostic value in evaluation of renal pathology even without the use of contrast agent. In the future it could be used to gain additional information of the tumor composition *ex vivo* after partial or total nephrectomy before histological workup. Unlike destructive histology that only provides two-dimensional information of representative and macroscopically suspicious regions, gbPC-CT could visualize the whole extent of the tumor in three-dimensions. Thus, introduction of a sample gbPC-CT scanner to clinical routine could help to speed up and elevate the certitude of the pathological evaluation. Furthermore, gbPC-CT could be used for tumor tissue characterization in *ex vivo* imaging studies, as previously shown for pancreatic cancer^29^ on animal models. Introduction of clinical phase-contrast CT scanners would benefit the diagnosis of RCCs. This could be also of future interest to differentiate benign lesions like oncozytomas or angiomyolipomas from RCCs or detect micrometastases in renal parenchyma in *in vivo* imaging additionally to CT or MRI. However, this step is not straightforward and requires major technical efforts.

One of the major technical challenges that would have to be overcome is the size of the field of view, which is currently severely limited by the size of the available gratings. However, recent advances in the field of grating production provide promise that this obstacle may be overcome^30^. Furthermore, phase-contrast CT requires acquisition of images with different relative grating positions^31^ which implies longer scan times. It has been reported that as few as two grating positions are enough to obtain the phase-contrast and the conventional absorption signal^32^. Thus, it means that the total scan time would increase by a little bit more than a factor of two compared to the currently used protocols. A scan time increase by a factor of two seems to be reasonable for renal imaging, though the risk of movement artifacts due to prolonged acquisition time increases. Moreover, in humans, the overlaying structures may complicate the acquisition of phase contrast. Especially such highly absorbing structures as the ribcage can cause severe image artifacts. Therefore, it is necessary to consider advanced iterative reconstruction algorithms as has been reported by Hahn *et al*.^33^. Further challenges that need to be resolved before the method can be translated to human imaging include the technical feasibility of placing a grating interferometer onto a very compact gantry, and its stability during very fast rotation.

Several studies^34^,^35^,^36^ have reported in the past that a clinCT scan performed at 80 kVp instead of 120 kVp could be beneficiary for the visualization of pathologies especially for thin patients. Therefore, future studies should seek to analyze how a 80 kVp clinCT scan could help better diagnose RCCs in patients and compare the results to gbPC-CT imaging.

Compared to attenuation-based CTs, gbPC-CT allowed for improved visualization of soft-tissue and tumor tissue architecture and improved discrimination of normal kidney and tumorous tissue of *ex vivo* samples of renal cell carcinoma subtypes without application contrast media with an excellent visual agreement to histopathology and MRI. GbPC-CT has the potential to improve renal imaging and may further be used during histopathological workup of large tumors to visualize diagnostically valuable tissue sections with a three-dimensional whole-sampling imaging.

## Material and Methods

### Samples

This retrospective experimental *ex vivo* study was approved by the local ethics committee (Ethikkomission der Universität München, München) and carried out in accordance to the international Declaration of Helsinki. Informed consent was obtained from all patients. Indication to partial or total nephrectomy followed recommendation of the interdisciplinary tumor conference. After surgical tumor excision, representative tumor tissue sections of 3 cm maximum diameter and 10 cm maximum length were selected by an experienced pathologist and put into 50 ml plastic containers in 4% formaldehyde solution.

In total, 23 human renal *ex vivo* samples were collected including 8 clear cell (ccRCC; 1x T2, 7x T3; mean diameter: 9 cm [5.8–16 cm]), 7 papillary (pRCC; 2x T1, 2x T2, 3x T3; mean diameter: 12 cm [5.5–23 cm]) and 5 chromophobe RCCs (chrRCC; 1x T1, 2x T2, 2x T3; mean diameter: 6.5 cm [3.2–10 cm]) as well as 3 healthy kidney sections after total nephrectomy.

### Grating-based X-ray imaging

The grating-based phase-contrast setup was described in detail elsewhere^37^. Attenuation-based (gbCT), phase-contrast (gbPC-CT) and dark-field computed tomographic images were simultaneously obtained^12^,^38^, the latter were not considered in this study^12^,^39^.

Samples were imaged without contrast media. Detailed scan parameters can be found in Table 2. The effective pixel size was 100 × 100 μm^2^. Three-dimensional reconstruction was done of each scan. For a better calibration of the quantitative HU and HUp values a circular PMMA rod was included in every sample^14^. Due to the highly experimental character of the imaging setup no speed or dose optimization was performed. The radiation dose for a full sample tomographic scan was 10–15 Gray. Images were recorded and stored as DICOM-data.

### Clinical CT and MR-imaging

Unenhanced scans were recorded using a 64-slice clinical CT scanner (clinCT; Optima CT660, GE Healthcare). For quantitative measurements, calibration materials like water, air and 70% ethanol were placed together with the samples in a small acrylic glass body phantom. Samples were measured with a tube voltage of 120 kVp and a tube current of 80–200 mAs with a slice thickness of 0.625 mm and a pitch of 0.531. The calculated effective pixel size was 400 × 400 μm^2^ at a diameter of the field of view of 21 cm at a matrix of 512 × 512. After filtered back projection, multiplanar reconstructions (coronal, sagittal) were calculated with a slice thickness of 1 mm and an increment of 0.7 mm.

The same samples were examined using a 3T MRI (MAGNETOM Skyra, Siemens Medical Solutions) placed in a 16-channel hand wrist coil. T1 and T2-weighted sequences with and without fat-saturation as well as susceptibility weighted images (SWI) were acquired in transverse and coronal directions without additional contrast media (Table 3).

### Histology

Formalin-fixed samples were cut into 5-mm slices and embedded in hot paraffin wax. Representative tissue sections were cut into 5 μm sample sections and stained with hematoxylin and eosin (HE) using standard protocols.

An average of 10–12 histological slices were produced for each sample. Histological workup was performed by an experienced pathologist (V.M.). The diagnoses of the RCC subtypes was made based on microscopic evaluation according to histopathological diagnostic guidelines^40^. The diagnosis of a chromophobe RCC required further application of immunochemistry (Hale’s staining). Additionally, tumor components were classified as calcification, necrosis, hyalinization, hemorrhage, fibrous strands, pseudocapsules and fat.

### Imaging and Quantitative analysis

All images were analyzed using a 32-bit image open access viewer (OsiriX 5.8; Apple Inc.) by one experienced radiologist (5+ years of experience) blinded to histopathological diagnoses.

First, sensitivity for the separation of normal kidney from tumor tissue was qualitatively and quantitatively evaluated. Second, images were manually matched to the corresponding histological slices (10–12 for each sample) using features such as fat, calcifications or tumor outline. The visual detection of calcifications, hemorrhage, pseudocapsules, fibrose strands, necrosis or hyalinization in the different imaging modalities corresponding to the matched histological slices as reference standard was assessed by the radiologist and pathologist in consensus. The sensitivity to detect tumorous tissue in the different imaging modalities was calculated as the fraction of detected tumors compared to the histological findings.

Third, Phase-contrast Hounsfield units (HUp) were quantified^14^,^20^ for all kidney samples (n = 23) by placing regions of interest (ROIs). In each sample, 10–12 ROIs were placed on gbPC-CT and clinical CT images either encircling the entire tumor or individual tissue components, excluding calcification areas or macroscopic fat. Delineation was done in comparison to the corresponding histological sections in consensus of the radiologist and pathologist. Quantitative values of normal kidney parenchyma were calculated by placing ROIs in cortical and medullary regions of healthy human kidney samples (n = 3) and normal kidney parenchyma adherent to RCCs (n = 6).

### Statistical Data Analysis

Statistical analysis was performed using SPSS (IBM SPSS 23.0, SPSS Inc). Mean values and standard deviations of HUp as well as independent t-tests were calculated using Excel (Excel 2008; Microsoft Inc.). A *p*-value of less than 0.05 indicated a statistically significant difference.

## Additional Information

**How to cite this article**: Braunagel, M. *et al*. Qualitative and Quantitative Imaging Evaluation of Renal Cell Carcinoma Subtypes with Grating-based X-ray Phase-contrast CT. *Sci. Rep.* **7**, 45400; doi: 10.1038/srep45400 (2017).

**Publisher's note:** Springer Nature remains neutral with regard to jurisdictional claims in published maps and institutional affiliations.

## Figures and Tables

**Table 1:** Detection of different tumor components in gbPC-CT, gbCT, clinCT and MRI compared with histopathologic findings.

	gbPC-CT/Histo	gbCT/Histo	clinCT/Histo	MRI/Histo
Fibrous strands	15/15	0/15	0/15	4/15
Pseudocapsule	8/9	0/ 9	0/9	6/9
Calcification	9/17	17/17	4/17	*
Microbleeding	10/11	0/11	0/ 11	*
Diffuse hemorrhage	5/5	3/5	2/5	5°/5
Hyalinization	6/8	6/8	2/8	6/8
Necrosis	2/3	0/3	0/3	1/3

Note. – detection in the different modalities was determined according to histopathological findings.

*Small susceptibility artifacts in susceptibility weighted images (SWI) in 17 samples.

°large hypointense areas.

gbPC-CT – grating-based phase-contrast computed tomography; gbCT – grating-based computed tomography; clinCT – clinical computed tomography; MRI – magnetic resonance imaging; Histo – histological findings.

**Table 2:** Technical parameters for grating-based X-ray imaging.

Voltage	40 kVp
Number of projections	800 over 360°
Number of phase steps	11
Exposure per phase step	3 s
Total scan time	11, 5 h for 2 cm of sample
Average sample height	6 cm (range 4 to 8 cm)

**Table 3:** Technical parameters for sample imaging in 3 T MRI.

Sequence	T1-w VIBE ± FS		T2-w TSE ± FS		SWI
Orientation	transverse	coronal	transverse	coronal	transverse
TR (ms)	11.5	12.83	6890	3620	27
TE (−FS/+FS) (ms)	3.69/4.92	4.41/5.64	44	59	20
FA	10°	10°	150°	150°	—
FOV (mm)	200	200	200	180	204
Voxel size (mm^3^)	0.4 × 0.4 × 1.0	0.3 × 0.3 × 0.7	0.5 × 0.5 × 1.0	0.4 × 0.4 × 1.0	0.8 × 0.8 × 2.0

VIBE – Volumetric interpolated brain examination; TSE – Turbo spin echo; FS – fat saturation; SWI – susceptibility weighted imaging; TR – repetition time; TE – echo time; FA – flip angle; FOV – field of view.
